# Editorial – Looking forward to a new year

**DOI:** 10.1017/ehs.2020.3

**Published:** 2020-01-24

**Authors:** Ruth Mace

**Affiliations:** London January 2020

## Abstract

**Figure:**
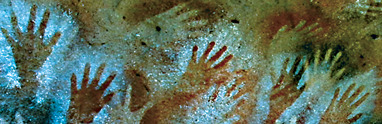

Writing from London, some of us are recovering emotionally from failing to stop Brexit. Let's hope the damage being done is short lived. Evolutionary human scientists have certainly made good use of the European Research Council, to give just one example of many useful things the UK may now lose (€95 million of research funding). We don't know if there will be any more of these grants after the end of next year. A couple of nights ago I was at an ‘End of 2019’ drinks party with some of the neighbours; what with Brexit and a long list of other disasters elsewhere in the world, the conversation was perhaps not as jolly as it should have been. One neighbour muttered gloomily ‘It's the end of the world really’. That might be overly pessimistic. I gather the UK is self-sufficient in cucumbers, so at least my pet tortoise will be fine. Let's hope 2020 gives us some reasons to cheer up.

I am actually very happy with how the journal is going. Since we started just over a year ago, we have had 89 submissions. Most are research articles, some are reviews, and we have recently opened a new category, methods papers. Some special collections are brewing. So far, 17 of those papers have made it into print. Don't panic – that does not mean we have an 80% rejection rate. Many more manuscripts are in various stages of review, revision or production, and some are registered reports so the science is underway. As I hoped, submissions have ranged right across the evolutionary human sciences spectrum, from the evolution of punishment, language, cognition, medical or various other cultural practices, to tracking migrations of East Asians using archaeology, linguistics and ancient DNA. We learnt, amongst many other things, that primate culture may be more widespread than we thought; Agta hunter-gatherers have extreme male-biased sex ratios at birth but natural mortality means that the bias is gone by adulthood; ultra-marathon runners pick their niche in line with Allen's rule, with the taller, thinner athletes more likely to compete in hot climates than in cold, and more likely to finish in hot conditions; people living in well-educated neighbourhoods are more helpful. I am delighted that you have trusted us with your work.

We want to be as author-friendly as possible, looking for reasons to accept rather than reject. Some papers are of course rejected (not yet ready, hypothesis or interpretation is unclear, etc.). The peer review process frequently improves papers dramatically, but sometimes it can feel like censorship. Crossing disciplinary boundaries can present challenges; an anthropologist, an economist and a biologist may all be interested in the same topic but have different ideas about the best way to do research. The peer review system is, like democracy, not perfect but hard to improve upon. We do our best to balance keeping all parties happy whilst promoting good science, but as I already knew from previous editorial experience, it seems *on ne saurait faire d'omelette sans casser des oeufs.*

Do suggest reviewers when you submit papers and we will try to use at least some of them (although the recommended reviewers are often no less hard-nosed than the others). Think twice before recommending ‘big names’; they may do their fair share of reviewing but can be bombarded with requests, so are less likely to be available. Think about recommending early-career researchers that editors may be less familiar with.

We aim for our science to be open and reproducible. I hope authors can make data and code available, when it is practicable. I do not usually expect reviewers to reanalyse the work (a task normally left to those who are interested after publication). Open science just means being transparent about your methods and your data. We are of course happy to publish ‘negative’ results if the hypothesis is of interest. Editorial judgement is still needed about which non-significant results to publish if the method or hypothesis is weak. Reanalysis, meta-analyses, pre-registration are all trying to raise the bar. A failure to replicate an erroneous conclusion hopefully redirects researchers to more fruitful avenues. However, it is rarely that simple; there are several vampire hypotheses that are seemingly impossible to kill. Often it may be the theory that is the problem. Biased data are another thorny issue that cannot easily be resolved by reanalysis. Science surely self-corrects in the end, and this journal will try to help that process, but there is no panacea. Thoughtful new ways of testing interesting hypotheses (new or old) are still needed just as much now as they ever were.

Open access publishing is becoming increasingly popular. It does not mean publication is cost free, however. At present Cambridge University Press is paying for the production of the journal (and editors and reviewers are giving their time mostly for free – thank you!). *EHS* will continue to not charge for one more year, and after that authors will pay, hopefully supported by their institutions or research funders. EHBEA members will get a good discount when we do eventually charge. Meanwhile make the most of 2020 to publish your work with us for free!

I look forward to meeting some of you online or in person on my travels over the coming year, many of you hopefully at EHBEA in Krakow in April. I wish you a Happy New Year, 新年快乐, Bonne Anné, and I hope it brings you all good things.

